# Novel Mechanism for Buffering Dietary Salt in Humans: Effects of Salt Loading on Skin Sodium, Vascular Endothelial Growth Factor C, and Blood Pressure

**DOI:** 10.1161/HYPERTENSIONAHA.117.10003

**Published:** 2017-10-01

**Authors:** Viknesh Selvarajah, Kaisa M. Mäki-Petäjä, Liliana Pedro, Sylvaine F.A. Bruggraber, Keith Burling, Anna K. Goodhart, Morris J. Brown, Carmel M. McEniery, Ian B. Wilkinson

**Affiliations:** From the Division of Experimental Medicine and Immunotherapeutics, University of Cambridge, United Kingdom (V.S., K.M.M-P., A.K.G., C.M.M., I.B.W.); MRC Human Nutrition Unit, Cambridge, United Kingdom (L.P., S.F.A.B.); NIHR Cambridge Biomedical Research Centre, Core Biochemical Assay Laboratory, United Kingdom (K.B.); and William Harvey Research Institute, Queen Mary University of London, United Kingdom (M.J.B.).

**Keywords:** blood pressure, skin, sodium, stroke volume, vascular endothelial growth factor C

## Abstract

High dietary sodium intake triggers increased blood pressure (BP). Animal studies show that dietary salt loading results in dermal Na^+^ accumulation and lymphangiogenesis mediated by VEGF-C (vascular endothelial growth factor C), both attenuating the rise in BP. Our objective was to determine whether these mechanisms function in humans. We assessed skin electrolytes, BP, and plasma VEGF-C in 48 healthy participants randomized to placebo (70 mmol sodium/d) and slow sodium (200 mmol/d) for 7 days. Skin Na^+^ and K^+^ concentrations were measured in mg/g of wet tissue and expressed as the ratio Na^+^:K^+^ to correct for variability in sample hydration. Skin Na^+^:K^+^ increased between placebo and slow sodium phases (2.91±0.08 versus 3.12±0.09; *P*=0.01). In post hoc analysis, there was a suggestion of a sex-specific effect, with a significant increase in skin Na^+^:K^+^ in men (2.59±0.09 versus 2.88±0.12; *P*=0.008) but not women (3.23±0.10 versus 3.36±0.12; *P*=0.31). Women showed a significant increase in 24-hour mean BP with salt loading (93±1 versus 91±1 mm Hg; *P*<0.001) while men did not (96±2 versus 96±2 mm Hg; *P*=0.91). Skin Na^+^:K^+^ correlated with BP, stroke volume, and peripheral vascular resistance in men but not in women. No change was noted in plasma VEGF-C. These findings suggest that the skin may buffer dietary Na^+^, reducing the hemodynamic consequences of increased salt, and this may be influenced by sex.

## Abstract 2

Supplemental Digital Content is available in the text.

Large population studies suggest that excessive dietary sodium, principally as the chloride salt, is an important trigger for hypertension.^1,2^ The mechanisms for this relationship are still debated.^3,4^ In the classical paradigm, increased salt intake leads to increased sodium accumulation in the extracellular space with a corresponding increase in extracellular volume, which can be partly counterbalanced by pressure natriuresis.^5^ However, more recent studies looking at sodium balance in humans have shown that large amounts of sodium can accumulate without commensurate water retention.^6–8^ These observations oppose the traditional view that sodium balance functions as a 2-compartment model, supporting the existence of nonosmotic sodium retention in a third compartment. In support of this, studies in rat models have shown that the skin is capable of osmotically inactive Na^+^ storage, via glycosaminoglycans (glycocalyx), serving as an important mechanism for buffering volume and blood pressure (BP) changes with salt intake.^9–11^ High-salt intake in rats also stimulates tonicity-responsive enhancer binding protein secretion by mononuclear phagocyte system cells, which mediates VEGF-C (vascular endothelial growth factor C) expression. This results in enhanced interstitial lymphatic drainage and increased expression of endothelial nitric oxide (NO) synthase, which buffers the hemodynamic response to salt loading.^10,11^ However, the relevance of these mechanisms in humans is unclear. In humans, the skin is the largest organ in the body, constituting 6% of body weight and receiving 20% to 30% of the cardiac output under normal conditions and up to 60% in erythroderma and other pathological conditions, and its role in BP control is of current interest.^12,13^ Recent ^23^Na magnetic resonance imaging (MRI) studies in humans show a direct relationship between skin Na^+^ and BP, as well as age and sex differences in the capacity to store skin Na^+^.^14,15^ Although MRI data were confirmed by direct ashing of human cadaveric samples, they have not yet been confirmed by direct chemical analysis of skin electrolytes in humans.^16^ Moreover, ^23^Na MRI has not been used to measure changes in skin Na^+^ with dietary salt modulation. We hypothesized that the degree of change in skin sodium would relate to the BP change seen with dietary salt loading. We tested this hypothesis in a group of young healthy adults in whom we studied change in skin Na^+^ with increased dietary salt intake, by direct chemical measurements. We also sought to study the correlation between skin sodium, BP, other hemodynamic variables, and plasma VEGF-C.

## Methods

### Subjects

Participants were healthy individuals aged between 18 and 50 years and recruited by advertisement. Exclusion criteria included hypertension (sustained BP >140/90 mm Hg), current use of antihypertensive drugs, diuretics, salt supplements, renal impairment, or pregnancy. All participants gave informed consent before study participation. Ethical approval for the study was obtained from a National Research Ethics Committee (REC Reference: 11/H0304/003) and was performed according to Good Clinical Practice and according to the principles of the Declaration of Helsinki.

### Study Protocol

We conducted a 4-week single-center, double-blind, randomized, cross-over study as depicted in Figure S1 in the online-only Data Supplement. Volunteers were screened and placed on a 4-g low-salt diet (equivalent to 70 mmol of sodium/d) at visit 1 to standardize the background sodium intake. Participants were outpatients and given a printed booklet provided by practicing dietitians on how to maintain a low-salt diet, with email and telephone advice offered throughout the duration of the study. Baseline salt consumption was assessed with 24-hour urinary sodium excretion (UNaV) up to 3 days after visit 1. Participants with high baseline salt intakes were given further advice on dietary salt restriction. After a 1-week run-in period on a low-sodium diet, participants received slow sodium (200 mmol/d for 7 days) or placebo tablets during weeks 2 and 4 in random order. Study compliance was assessed by 24-hour UNaV within 48 hours of visits 2, 3, and 4 with participants and investigators blinded to the results. Medication compliance was assessed by participants returning used tablet bottles. Each participant was seen at approximately the same time at each study visit in a temperature-controlled room. Participants were required to refrain from caffeine, alcohol, strenuous exercise, and the application of moisturizer or fake tan to their lower back for 6 hours before study visits. At each visit, weight was measured using the same calibrated scales, with 1 layer of clothing on no shoes, and seated brachial BP was recorded after a minimum of 5 minutes rest. After a further 10 minutes supine rest, brachial BP, cardiac output, stroke volume, pulse wave analysis, and aortic pulse wave velocity were taken. Ambulatory BP monitoring was recorded with 24-hour UNaV collections within 48 hours of visits 2, 3, and 4. At visits 2 and 4, after hemodynamic measurements, a skin biopsy was taken. The skin biopsies were taken from the lower back to minimize any impact of scarring. The first biopsy was done on the right and the second on the left in a symmetrical position. Skin was analyzed by inductively coupled plasma optical emission spectrometry (ICP-OES) to determine Na^+^ and K^+^ concentrations in milligrams per gram of wet skin. ICP-OES is a highly sensitive analytic tool capable of simultaneous multielemental determinations down to the subparts-per-billion level.^17^ We summarize the technique in the online-only Data Supplement (Figures S2 and S3), together with the specific methods for hemodynamic and biochemical measurements and skin biopsies.

### Statistical Analysis

The primary outcome measure was change in skin sodium concentration between the placebo and slow sodium phases. Because of technical limitations of drying small tissue samples, encountered when validating the ICP-OES, we had to change our measure from absolute dry Na^+^ to Na^+^:K^+^ ratio. As published data available on changes in skin sodium concentration in response to salt loading in humans were lacking, the initial sample size of 48 was based on other salt loading studies of a similar design, with a planned interim analysis to assess effect size and power. Normally distributed data are presented as mean±SEM and non-normal data as median and interquartile range. Tests for normality were performed using the Shapiro–Wilk test. Student paired *t* tests were applied to paired observations after placebo and slow sodium for normally distributed data and the Wilcoxon signed-rank test for non-normally distributed data. Independent samples *t* tests were applied to unpaired observations (normally distributed data) and the Mann–Whitney test (non-normally distributed data). Correlation coefficients between skin Na^+^:K^+^ and putative parameters, such as age, sex, body mass index, body surface area, and hemodynamic variables, were calculated using Pearson method (normally distributed variables) and Spearman method (non-normally distributed variables). We then performed multiple regression analysis to examine the parameters that independently influence skin Na^+^:K^+^. In analyzing the data, it became apparent that that skin Na^+^:K^+^ differed by sex, and a post hoc sex-specific analysis was therefore undertaken. The presence of carry-over effect was checked using univariate ANOVA. A probability of <5% was used to reject the null hypothesis. Statistical analysis was performed with SPSS software (version 23.0).

## Results

**Figure 1.**
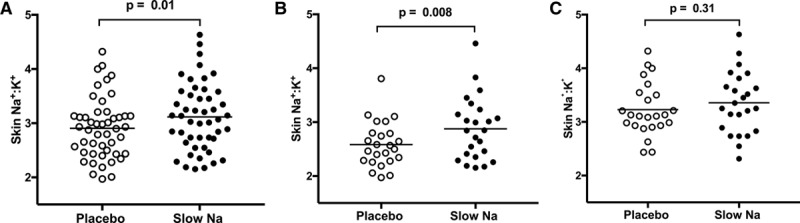
Changes in skin Na^+^:K^+^ ratios between placebo and slow sodium phases. **A**, All 48 participants. **B**, 24 males. **C**, 24 females. The change in skin Na^+^:K^+^ between placebo and slow sodium for males and females, respectively, was analyzed using the Student paired *t* test. *P*<0.05 taken to be significant.

In all, 24 men and 24 women completed the study (mean age of 30±2 years; range, 18–49 years). Baseline characteristics are shown in Table S1. All women were pre-menopausal, and 10 were using an oral contraceptive pill or contraceptive implant. Our study population had a lower baseline sodium intake compared with the current average intake in England (≈130 mmol/d).^18^ The primary end point was the change in skin Na^+^:K^+^ between placebo and slow sodium phases. There was an increase in skin Na^+^:K^+^ between placebo and slow sodium phases (2.91±0.08 to 3.12±0.09; *P*=0.01; Figure 1A). In multiple regression analysis, including age, body mass index, clinic mean arterial pressure, and sex, only sex remained independently associated with skin Na^+^:K^+^ values (*R*^2^=0.316; *P*<0.001). This was primarily because in women, skin K^+^ was lower and the Na^+^:K^+^ ratio was consequently higher. Therefore, subsequent analyses were sex specific. Skin Na^+^, K^+^, and Na^+^:K^+^ values are presented in Table 1 and Figure 1. Men showed a significant increase in skin Na^+^:K^+^ of 11.2% (*P*=0.008) from placebo to slow sodium phases while women showed nonsignificant increase of 4.0% (*P*=0.31). However, there was no significant difference between sexes on formal testing (repeated measures ANOVA *P*=0.31). Neither sex showed a significant change in skin K^+^ with a change in salt intake, in keeping with previous animal studies.^19,20^ No evidence of carry-over effect was seen for skin Na^+^:K^+^ or skin Na^+^ for either sex using univariate ANOVA with order of treatment (placebo or slow sodium) as a fixed factor (*P*=0.68).

**Table 1.**
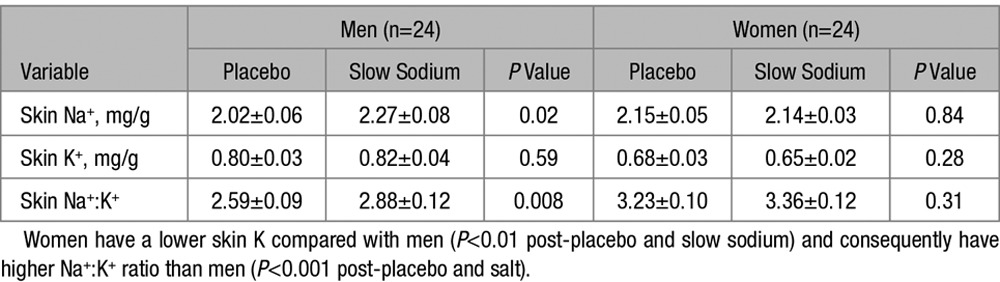
Differences in Skin Biochemical Responses to Placebo vs Slow Sodium by Sex

Hemodynamic responses to placebo versus slow sodium are shown in Table 2. In men, there were no differences in hemodynamics after salt loading while women had higher 24-hour systolic BP, 24-hour mean arterial pressure, daytime systolic BP, nighttime systolic BP, diastolic BP, and mean arterial pressure. Only women had an increase in body weight post-slow sodium (63.5±1.6 versus 64.2±1.6 kg; *P*=0.01). Baseline characteristics and hemodynamic responses to salt loading were similar in women with and without contraceptive use (Tables S3 and S4). Differences in biochemical responses to placebo versus slow sodium are shown in Table 3. As expected, slow sodium increased 24-hour UNaV, serum Na^+^, and Cl^−^ and suppressed plasma renin and aldosterone. There were no significant changes in plasma VEGF-C levels or sFLT-4 (soluble fms-like tyrosine kinase 4, a soluble receptor for VEGF-C) between placebo and slow sodium in either sex or in the whole study population.

**Table 2.**
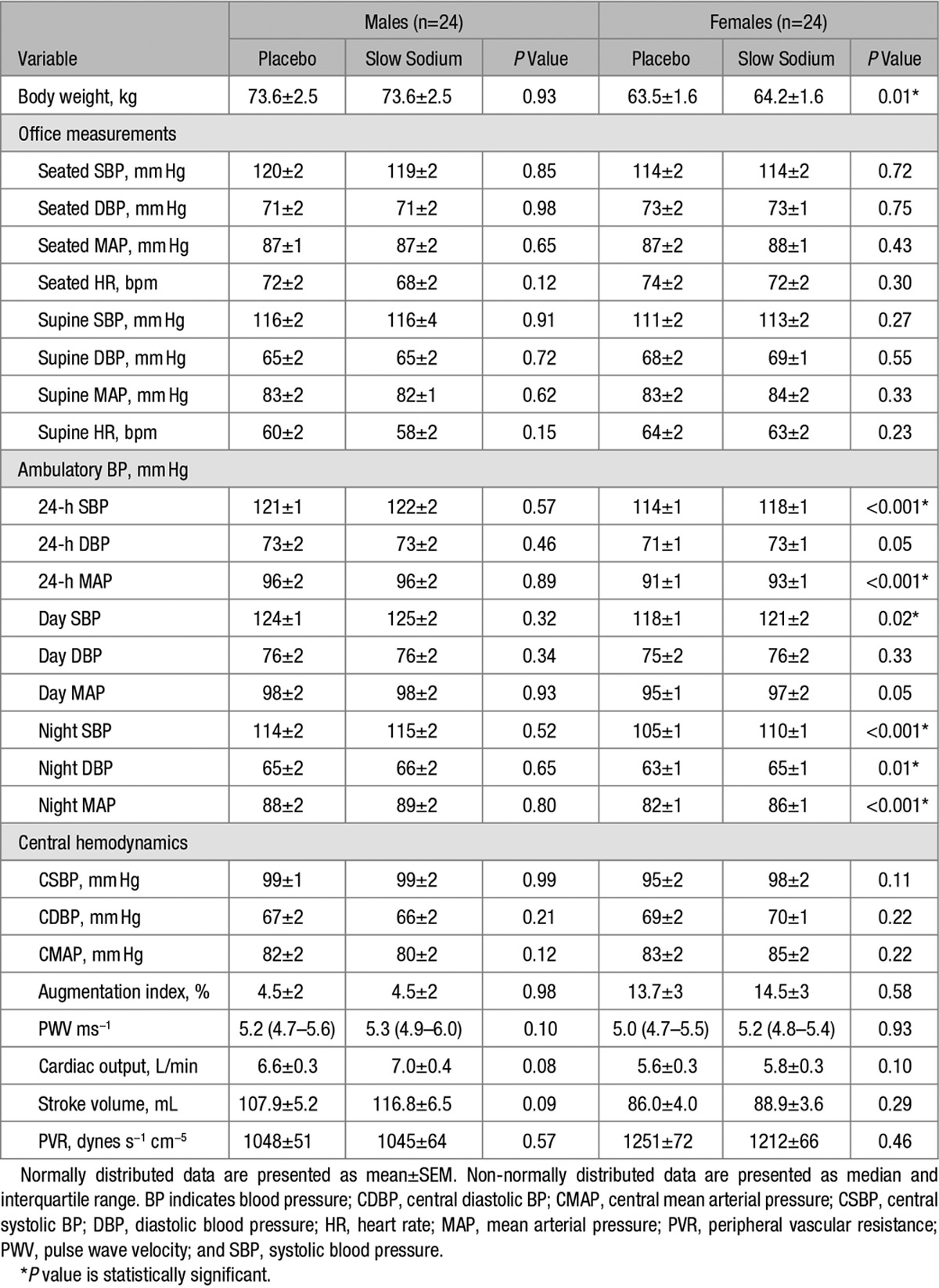
Differences in Hemodynamic Responses to Placebo vs Slow Sodium by Sex

**Table 3.**
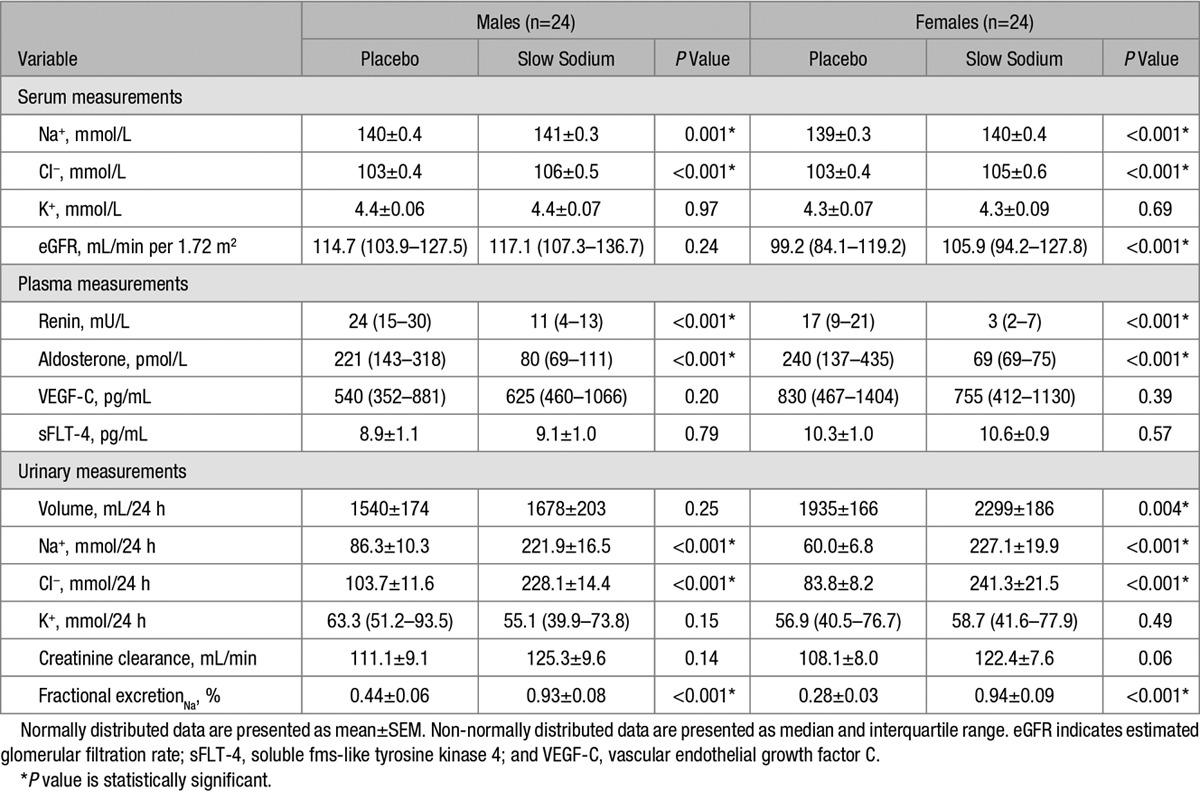
Differences in Biochemical Responses to Placebo vs Slow Sodium by Sex

**Figure 2.**
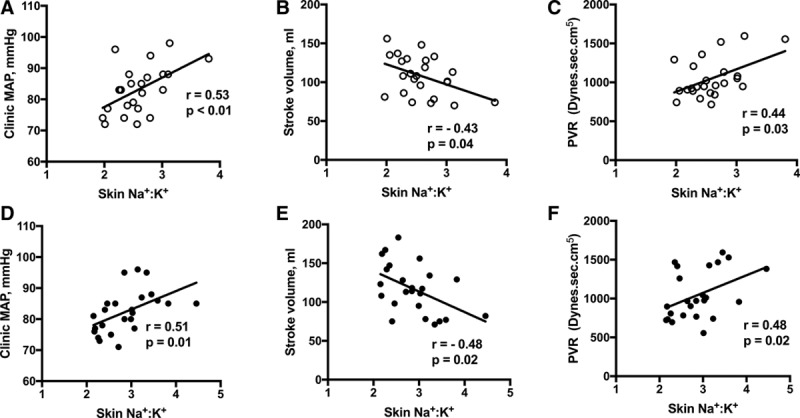
Correlation between skin Na^+^:K^+^ and haemodynamic variables in 24 male participants. **A**, Supine brachial mean arterial pressure (MAP) post-placebo. **B**, Stroke volume post-placebo. **C**, Peripheral vascular resistance (PVR) post-placebo. **D**, Supine brachial MAP post-slow sodium. **E**, Stroke volume post-slow sodium. **F**, PVR post-slow sodium.

**Figure 3.**
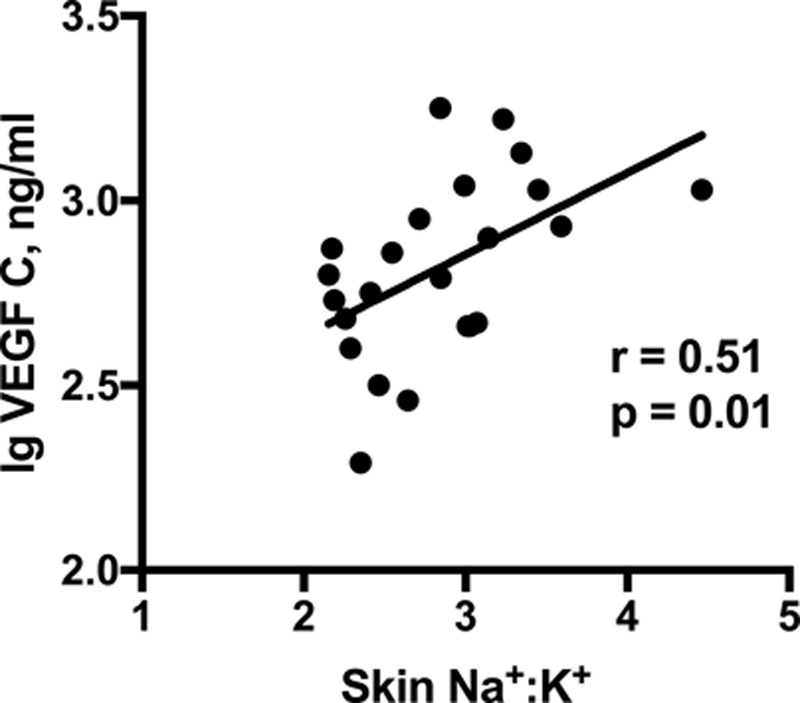
Correlation between skin Na^+^:K^+^ and VEGF-C (vascular endothelial growth factor C) post-slow sodium in 24 male participants.

Univariate analyses revealed significant correlations between skin Na^+^:K^+^ and hemodynamic variables in men only as shown in Figure 2. Skin Na^+^:K^+^ correlated positively with supine mean arterial pressure post-placebo (*r*=0.53; *P*<0.01) and post-slow sodium (*r*=0.51; *P*=0.01). We observed that skin Na^+^:K^+^ was negatively correlated with stroke volume and positively correlated with peripheral vascular resistance (PVR) post-placebo and slow sodium. Plasma VEGF-C, after logarithmic transformation, showed a positive correlation with skin Na^+^:K^+^ post-slow sodium (*r*=0.51; *P*=0.01) but not placebo (*r*=0.06; *P*=0.79; Figure 3). All correlations were independent of age and body mass index on multiple regression analysis. Correlations between hemodynamic parameters and skin Na^+^:K^+^ in women and urine Na^+^:K^+^ are shown in Figure S6 and Table S5, respectively.

## Discussion

The main findings of this study are that skin sodium increases with dietary salt loading, and this may be influenced by sex. The dietary salt load we used (200 mmol Na^+^ for 7 days) is within the normal daily intakes of many individuals in urban societies and thus would be clinically relevant.^2^ The primary end point was significant, with skin Na^+^:K^+^ increasing between the placebo and slow sodium phases for the whole study population, but in our study, this appeared to be mainly driven by men.

Our measurements show that women have lower skin K^+^ and consequently a higher Na^+^:K^+^ ratio (Table 1). The epidermis has a significant K^+^ content, and this difference could be explained by the epidermis being thinner in women.^21,22^ Therefore, changes in Na^+^:K^+^ ratio with salt loading can be compared, but it is not possible to directly compare values for skin Na^+^:K^+^ between men and women. The use of contraceptives appeared to influence skin K^+^ values (Table S2). The reasons for this are unclear although estrogen administration increases epidermal thickness.^23^ Importantly, women have the same response in skin Na^+^:K^+^ with salt loading regardless of contraceptive use (Table S2 and Figure S5).

Previous human data on skin electrolytes are sparse. Oral salt loading experiments conducted over 80 years ago, in a small number of humans, showed that 10 g NaCl administered daily for 1 week increased the Na^+^ content of human skin by 15% to 20%, suggesting the skin was a repository for NaCl.^24^ Direct chemical measurements in human skin, performed >70 years ago, yielded values of between 1.00 to 2.14 mg/g for Na^+^ and 0.64 to 0.91 mg/g for K^+^ per wet weight of skin.^25–27^ Our values for Na^+^ and K^+^ fell within this range but are difficult to compare with skin Na^+^ measured using MRI, which are expressed as mmol/L of water.^14,16^ Elemental measurements per wet weight of tissue may have potential inaccuracies introduced by changes in sample hydration during sample processing, which is why previous animal studies expressed skin Na^+^ and K^+^ concentrations per unit dry weight after dry ashing.^11,19,20^ Our samples had an average wet weight of ≈15 mg compared with rat skin samples (≈60 g) and, unfortunately, were not suitable for dry ashing. Because our freeze-drying technique could not remove moisture uniformly in small samples, we corrected for sample hydration by expressing skin Na^+^ as a ratio Na^+^:K^+^ because of our ability to measure K^+^ reliably with ICP-OES and evidence from animal work that skin K^+^ remains stable with extremes of salt intake.^11,19,20^ Importantly, skin K^+^ did not change with salt loading. Furthermore, the Na^+^ content of injected local anesthetic did not seem to contaminate our skin samples (Methods in the online-only Data Supplement; Figure S4).

In our post hoc analyses, skin Na^+^:K^+^ differed between men and women after placebo and slow sodium, and it appeared that skin Na^+^:K^+^ rose with salt loading in men but not in women. Although this sex difference failed to achieve significance with formal ANOVA comparison, no doubt because of the small sample size, it is potentially interesting. Women had a significant increase in day and night ambulatory BP while men did not. This was associated with significant weight gain in women, in keeping with previous data demonstrating greater sensitivity to dietary sodium in women.^28–30^ Baseline sex differences in hemodynamic and biochemical variables were also in keeping with previous studies in healthy young adults.^31–35^ Augmentation index is higher in women, and this is thought to be because of women having shorter stature and higher PVR when corrected for body surface area.^33,36^ Our study was not powered to show a sex difference in PVR or pulse wave velocity. There could be several explanations for the sex differences observed with salt loading.

The reason for observed sex difference in salt sensitivity is unclear, and the importance of addressing this issue has been highlighted recently.^3,4^ In our study, there was a modest, but statistically significant difference in 24-hour UNaV, where women were lower than men post-placebo, suggesting that women were either more adherent to a low-salt diet or simply ate less. This difference was statistically significant but modest. There are known limitations of using 24-hour UNaV to estimate Na^+^ intake, with recent evidence showing ±25 mmol deviations in urinary Na^+^ from recorded Na^+^ intake.^37^ An alternative explanation is that BP in men did not change with a short-term changes in salt intake because they could buffer the additional dietary Na^+^ with their skin via mechanisms described in animal studies while in women, this ability was attenuated.

In animals, the skin buffers dietary salt, and a lower capacity to store Na^+^ in the skin is associated with a greater BP rise during acute salt loading.^38^ Recent evidence in humans suggests that the ability to store Na^+^ in the interstitium serves as a protective factor against the pressor effect of salt.^30^ Our results are consistent with these data albeit with a sex difference. We propose 2 reasons why the sex differences may have been observed. First, men have a thicker skin at all ages and may have higher levels of dermal glycosaminoglycans.^39,40^ This could imply that the skin is a more effective buffer for dietary Na^+^ in men. Indeed, animal data demonstrate that with a high-salt intake, male rats have a higher capacity for osmotically inactive skin Na^+^ storage compared with fertile female rats.^38^ Second, comparison of skin Na^+^ measurements not corrected for differences in sample hydration, reveals a trend for women to have a higher skin Na^+^ post-placebo than men. This suggests that men had greater passage of Na^+^ through the skin than women rather than greater storage of skin Na^+^, and this passage of Na^+^ was protective in short-term salt loading. As shown in animal studies, the passage of skin Na^+^ into the skin would have resulted in efflux of Na^+^ via VEGF-C–mediated lymphangiogenesis, which relates to NO production by VEGF-C.^10,11^ This is consistent with the known adaptation of salt-resistant subjects to a salt load, which is vasodilation concomitant to the increase in cardiac output.^30,41–43^

We found significant correlations between skin Na^+^:K^+^ and various hemodynamic parameters in men, which were present regardless of salt intake. This suggests a physiological role for skin Na^+^ or Na^+^:K^+^ ratio in regulating normal hemodynamics. The negative correlation between skin Na^+^:K^+^ and stroke volume could reflect increased osmotically inactive Na^+^ binding to glycosaminoglycans, allowing Na^+^ without commensurate water accumulation and therefore a less pronounced rise in circulatory volume.^44^ The positive correlation between BP and Na^+^:K^+^ support recent ^23^Na MRI data, which showed a positive correlation between BP and skin Na^+^.^14,15^ The mechanisms for these observations remain to be explained but could potentially involve interactions between hypoxia-inducible transcription factors, NO, and skin Na^+^. The skin is a rich source of NO, and the expression of NO is altered by hypoxia-inducible transcription factors, which is in turn modulated by salt intake.^13,45,46^ This interaction could potentially modulate PVR and the resulting BP. Alternatively, dermal capillary rarefaction, which refers to a reduction in capillary blood flow and increase in PVR, is associated with increased dietary salt and hypertension in humans.^47^ These mechanisms could potentially explain the positive correlation between skin Na^+^ and BP, as well as PVR although, clearly, further studies are required. A distinction should be made between the role of skin Na^+^ in influencing BP in response to short- and long-term high-salt intake. In short-term salt loading, as in our study, dermal Na^+^ storage may buffer the BP response to salt. However, in the long term (months), the ability to maintain normal BP in the face of high-salt intake is dependent on the ability to decrease PVR.^48^ The positive correlation seen between skin Na^+^:K^+^ and PVR suggests that skin Na^+^ accumulation may, in the longer term, lead to higher BP by increasing PVR. This is supported by cross-sectional ^23^Na MRI data showing that skin Na^+^ storage higher in hypertensives.^14^ In women skin, Na^+^:K^+^ was positively correlated with PVR post-salt, but this was not independent of age. There was a trend for skin Na^+^:K^+^ to be lower in women on contraceptives. Therefore, the Na^+^:K^+^ ratio may have been less reliable for assessing correlations in women in our study.

We did not observe significant changes in plasma VEGF-C or sFLT-4 between placebo and slow sodium phases. A previous study looking at plasma VEGF-C in healthy adults noted no change on dietary salt loading.^49^ As far as we are aware, this was the first study to examine plasma sFLT-4. We did not measure skin VEGF-C levels and therefore cannot draw any conclusions on skin VEGF-C response. However, plasma VEGF-C in men correlated positively with skin Na^+^:K^+^ post sodium, suggesting that in the salt-loaded state, skin Na^+^ induced VEGF-C production, as seen previously in animal studies.^10,11^

This study has several limitations. The study size was small, but we used a state-of-the art technique to measure skin Na^+^, ICP-OES, which is more sensitive than ^23^Na MRI. Our participants were nonresident, and the control of Na^+^ intake was challenging, especially in men, most likely because of high salt levels in processed foods, which even highly motivated participants would have struggled to avoid during the 4-week study period.^50^ We could not normalize dietary Na^+^ intakes for body weight. Participants also did not have their K^+^ and calorie intakes strictly controlled. Women were not salt loaded on the same day of the menstrual cycle. Our skin biopsies were small, and we had no means of ascertaining whether they were representative of the whole skin or whether skin Na^+^ varies with time. We did not quantify glycosaminoglycans in our skin samples or show how much of this Na^+^ was osmotically inactive. We also could not measure Cl^−^ with ICP-OES. A further limitation was that we performed post hoc analysis by sex, and this was because of unexpected sex differences in skin K^+^ and the consequent limitations of using the Na^+^:K^+^. The sex differences in BP and skin Na^+^ are interesting but warrant further investigation and confirmation in larger studies. Our study was not primarily designed to determine salt sensitivity: it examined skin Na^+^ in response to changes in dietary Na^+^. Our target Na^+^ intake during the placebo phase was 70 mmol, which is high compared with other studies examining salt sensitivity.^28,29^ The use of ambulatory BP monitoring made the detection of significant BP changes in women possible. Similar studies should also be conducted in older people, other ethnicities, and hypertensives. We conducted a short-term study because salt loading during a longer period may not have been ethical and chronic skin electrolyte changes in response to changes in salt intake may be different. Despite these limitations, we were able to provide a unique insight into the influence of interstitial Na^+^ on the hemodynamic response to dietary salt and will inform further studies on skin Na^+^.

## Perspectives

Worldwide, hypertension has a significant prevalence and associated morbidity. An elevated BP is the single most important cause of cardiovascular disease, being responsible for 62% of strokes and 49% of coronary heart disease.^51^ Excessive salt intake is thought to play a crucial role in this epidemic.^52^ Our understanding of how salt affects BP and how we handle Na^+^ is lacking, and the traditional nephrocentric view of sodium and fluid balance provides an incomplete explanation for this phenomenon. Our study provides novel data on how skin Na^+^ is altered by dietary salt, influences systemic hemodynamics, and may influence the regulation of salt sensitivity in a sex-specific manner.

## Acknowledgments

VEGF-C and sFLT-4 assays were performed by the National Institute for Health Research (NIHR) Cambridge Biomedical Research Centre, Core Biochemical Assay Laboratory. Dr Paul Norris and Dr Maysoon Elkhawed contributed to the pilot study included in the online-only Data Supplement.

## Sources of Funding

V. Selvarajah was funded by the British Heart Foundation (grant number FS/12/33/29561) and NIHR. I.B. Wilkinson is funded by the British Heart Foundation. I.B. Wilkinson, C.M. McEniery, and K.M. Mäki-Petäjä receive Cambridge NIHR Biomedical Research Centre–Comprehensive Clinical Research Network support. The Human Research Tissue Bank is supported by the NIHR Cambridge Biomedical Research Centre. We thank the Medical Research Council (grant number MC_US_A090_0008/Unit Programme number U1059) for their support.

## Disclosures

None.

## Supplementary Material

## Novelty and Significance

### What Is New?

- Skin Na^+^ increases with dietary salt loading in humans.
- The skin may buffer dietary Na^+^, reducing the hemodynamic consequences of increased salt. This may be influenced by sex.
- Skin Na^+^ may influence blood pressure, stroke volume, and peripheral vascular resistance.

### What Is Relevant?

- Excessive salt intake is thought to play a crucial role in the development of hypertension, but the exact mechanisms have not been fully elucidated.
- Sex differences have been observed in the hemodynamic response to dietary salt, with some studies showing women have a greater blood pressure response to dietary salt modulation. This has still not been explained.

### Summary

Skin Na^+^ is influenced by dietary salt and influences systemic hemodynamics. It may influence the regulation of salt sensitivity in a sex-specific manner.
