# Supplementary material for: Novel Mechanism for Buffering Dietary Salt in Humans: Effects of Salt Loading on Skin Sodium, Vascular Endothelial Growth Factor C, and Blood Pressure
Source: Hypertension. 2017 Oct 1;70(5):930–7. doi: 10.1161/HYPERTENSIONAHA.117.10003 (PMC5640984; doi:10.1161/HYPERTENSIONAHA.117.10003)

**Online supplement**

**A novel mechanism for buffering dietary salt in humans: Effects of salt loading on skin sodium, VEGF-C and blood pressure.**

Viknesh Selvarajah^1^, MBChB, Kaisa M Mäki-Petäjä^1^, PhD; Liliana Pedro^2^, MSc; Sylvaine FA Bruggaber^2^, PhD; Keith Burling^3^, MPhil, Anna K Goodhart^1^, MB BChir, Morris J Brown^4^, MD, FRCP, FMedSci, Carmel M McEniery^1^, PhD; Ian B Wilkinson^1^, FRCP, DM

1. Division of Experimental Medicine and Immunotherapeutics, University of Cambridge, Cambridge, U.K.
2. MRC Human Nutrition Unit, Cambridge, U.K.
3. NIHR Cambridge Biomedical Research Centre, Core Biochemical Assay Laboratory, Cambridge, U.K.
4. William Harvey Research Institute, Queen Mary University of London, UK

**Correspondence:** Dr Viknesh Selvarajah, Experimental Medicine and Immunotherapeutics, University of Cambridge, Addenbrooke’s Hospital, Box 98, Cambridge CB2 0QQ, U.K. Tel: +44 1223 216895, Fax: +44 1223 216893, Email: [vs321@cam.ac.uk](mailto:vs321@cam.ac.uk)

**Methods**

**Hemodynamic measurements**

Brachial blood pressure was measured with the subject seated with the arm supported and resting supine, using a validated semi-automated oscillometric device (HEM-705CP, Omron Corporation), according to British Hypertension Society guidelines. All measurements were taken in triplicate and mean values used in the subsequent analyses. Ambulatory blood pressure monitoring (ABPM) was carried out within 48 hours of each visit using Mobil-O-Graph**®** (IEM, USA). The ABPM device was placed on the non-dominant arm. Ambulatory BP recordings were analysed with the Mobil-O-Graph**®** system software package. Cardiac output (CO) and stroke volume (SV) were determined by a non-invasive, inert gas rebreathing technique, which has previously been validated against thermodilution and direct Fick methods for measurement of pulmonary blood flow and cardiac output.^1-5^ While resting in supine position, subjects continuously rebreathed a gas mixture (1% SF_6_, 5% N_2_O, and 94% O_2_) over 20 seconds with a respiratory rate of 20 breaths/min. Continuous samples of expired gases were analysed by an infrared photoacoustic gas analyser (InnoCor; Innovision A/S, Chicago, IL, USA) to determine CO and SV. Peripheral vascular resistance (PVR) was calculated using supine mean arterial pressure within 1 hour of Innocor measurements as follows:

**PVR (dynes s^-1^cm^-5^) = Mean arterial pressure (mmHg) x 80/cardiac output (l min^-1^)**

Radial artery waveforms were measured using a high-fidelity micromanometer (SPC- 301; Millar Instruments) from the wrist of the non-dominant arm with the participant lying supine after a further five minutes of rest, as previously described.^6^ Pulse wave analysis (SphygmoCor, AtCor Medical) was used to calculate augmentation index (Aix), the difference in pressure between the first and second systolic peaks of the pulse wave, expressed as a percentage of the pulse pressure. Heart rate was recorded during measurement of the radial artery waveform and central mean arterial pressure (MAP) was obtained as the value given by the software derived from integration of the waveform. The aortic pulse wave velocity (aPWV) was measured using the same device by sequentially recording ECG-gated carotid and femoral artery waveforms, as described previously.^6^ The path-length for the determination of aPWV was measured as the surface distance between the suprasternal notch and femoral site minus the distance between the suprasternal notch and carotid site, using a tape measure. All measurements were made in triplicate by a single trained investigator and the mean values were used in the subsequent analysis.

**Biochemical measurements**

Serum and plasma were collected for electrolytes, (Na^+^, K^+^, Creatinine, renin, aldosterone) and estimated glomerular filtration rate. For each participant, a single EDTA sample was centrifuged at 4°C (3200 rpm for 15 minutes), and the plasma separated and stored at - 80°C prior to analysis for VEGF-C and sFLT-4 (soluble receptor for VEGF-C) by sandwich immunoassay. For VEGF-C we used Quantikine ELISA kits from R&D Systems, Abingdon, United Kingdom. sFLT-4 was measured by an in-house electrochemical luminescence immunoassay on the MesoScale Discovery (Rockville, MA, USA) assay platform using antibodies and standards from an R&D Systems DuoSet at the Core Biochemical Assay Laboratory (CBAL) in Cambridge. The 24-hour urine collections were made for Na^+^, K^+^ and Cl^-^ within 48 hours of each visit, starting at 08:00 and discarding the first morning void. Serum and urine electrolytes were measured in an accredited laboratory (Cambridge University Hospitals Department of Clinical Biochemistry).

**Skin biopsy procedure**

Skin punch biopsies (5mm diameter) were taken from the lower back, using local anaesthetic (lidocaine), on day 7 of slow sodium and placebo tablets by a single trained investigator. The skin was cleaned using 2% chlorhexidine gluconate solution, which was found to be Na^+^ and K^+^ free using ICP-OES. Any fat obtained with the biopsied tissue was removed. Skin samples were placed in cryovials and snap frozen in liquid nitrogen once they were obtained and then stored at −80°C until analysis.

**Pilot work for skin biopsy procedure and assessment of Na^+^ contamination**

We quantified conventional lidocaine (Xylocaine 1% with adrenaline, AstraZeneca) as having > 113 mmol/l Na^+^ with no recordable K^+^, thus raising the possibility of Na^+^ contamination during lidocaine administration. We developed a deep injection technique into the subcutaneous layer to avoid the introduction of lignocaine Na^+^ into the dermis in a pilot study of 37 healthy volunteers that was used for method development (Figure S2). The age range was 19 – 67 years and 19 were male. The skin biopsies were taken from the lower back and analyzed for Na^+^ using ICP-OES, as in the main study. Ethical approval for this pilot study was obtained as part of our main study (REC Reference: 11/H0304/003). We also checked skin Na^+^ values in skin tissue samples obtained from breast reduction surgery in 17 patients without local anesthetic to see if our pilot skin values suggested Na^+^ or water contamination. All breast skin tissue acquisition procedures and experimental protocols were approved by Cambridge University Hospitals Human Research Tissue Bank, under the generic ethics approval (LREC 11/EE/0011). We measured % weight change for both sets of samples with freeze drying to estimate % water content. As seen in Table S6, Na^+^ and % water values were similar for our pilot samples and breast reduction samples. We then carried out our main study. After we completed 32 people in our main study, we developed Na^+^-free lidocaine using Dextrose as an excipient (Lidocaine 1% with Dextrose 3.5%, Tayside Pharmaceuticals, Dundee), which was used in the remaining 16 participants. We compared skin Na^+^:K^+^ results obtained using both types of lidocaine and found they were similar (Figure S7).

**Skin elemental analysis**

**Instrument**

The skin elemental concentrations of Na and K were determined at the MRC Human Nutrition Research Unit (Cambridge, UK) using an ICP-OES (Jobin Yvon Horiba – ULTIMA 2C) equipped with a concentric PFA micro-flow nebulizer (0-2ml/min sample flow rate), a 50ml glass cyclonic spray chamber and a radial torch with an 3mm internal diameter (i.d.) alumina injector. Sample solutions were introduced from an auto-sampler (Jobin Yvon Horiba AS500) using a sample probe with 0.25mm i.d. sample tubing and 0.38mm i.d. pump tubing (orange/green). Instrument operating conditions are listed in Table S8. Strontium, added during sample preparation, was measured alongside Na and K to control for possible error in sample dilutions and/or uptake. Peak profiles were used to measure individual elements as described in table S6.

**Sample preparation**

The skin samples were weighed in a pre-weight polypropylene vial to determine their wet weights and then freeze- dried overnight (Mini Lyotrap, LTE scientific, Greenfield, Oldham, UK) until they reached a constant weight (dry weight). The vials were re-weighed after drying to determine water content. The dried materials were digested directly in the vial by adding a digestion solution containing 1:1 (volume for volume) mixture of (69%) HNO_3_ and (40%) H_2_O_2_. The volume of the digestion solution was adjusted to the sample dry weight to ensure complete digestion (e.g. for a 7mg sample dry weight, 150 μL digestion solution was added)_._ The samples were incubated overnight at room temperature followed by a second incubation overnight in a water bath at 40⁰C. More digestion solution was added if solution was not clear and the incubation at 40⁰C repeated. All samples were digested to completeness before being diluted 1:40 with ultra-high purity (UHP) water containing strontium (Sr, 1ppm final concentration). Digestion blank controls (i.e. empty vials) were prepared alongside the skin samples and analyzed collectively with the digested skin samples. (Figure S3) After the analysis was completed we noted a variability in sample drying, with smaller samples recording greater proportional water contents. For this reason Na^+^ values expressed as Na^+^:K^+^ ratios to correct for this variation in sample weight.

**Analysis**

A series of external calibration standards were prepared from 1000 ppm commercial stock solutions in 2% HNO3 (Na, Fluka Tracepure; K, Perkin-Elmer Pureplus), with final concentrations per element ranging from 0 to 15 ppm in a diluent matched to the digested solution (final concertation 0.86% HNO_3_, 1ppm Sr). The Na and K concentrations were calculated against the linear regression obtained from the calibration standards. Drift check solutions and blank (diluent) solutions were measured after every block of approximately 6 samples.

Matrix effect was corrected using pooled sample-based standards (PSBS) as previously described.^7^ Matrix effect is defined as the combined effect of all components of the sample on the measurement of the analyte.^8^ Briefly, to measure matrix effect, a pooled sample is created from the skin digest samples and aliquoted and spiked with Na and K with final concentrations per element ranging from 0 to 15 ppm and maintaining a sample dilution of 1:40. The slopes of the linear regressions obtained for each elements are compared with the slopes of the external calibration linear regression in diluent described above and a correction factor calculated. Skin elemental concentrations were expressed in matrix corrected milligrams of element per gram of dry or wet sample mass and as a Na:K ratio.

**References**

1. Clemensen P, Christensen P, Norsk P, Grønlund J. A modified photo- and magnetoacoustic multigas analyzer applied in gas exchange measurements. *J Appl Physiol.* 1994;76:2832–2839.

2. Peyton PJ, Thompson B. Agreement of an Inert Gas Rebreathing Device with Thermodilution and the Direct Oxygen Fick Method in Measurement of Pulmonary Blood Flow. *J Clin Monit Comput.* 2004;18:373–378.

3. Peyton PJ, Bailey M, Thompson BR. Reproducibility of cardiac output measurement by the nitrous oxide rebreathing technique. *J Clin Monit Comput.*2009;23:233–236.

4. Gabrielsen A, Videbaek R, Schou M, Damgaard M, Kastrup J, Norsk P. Non-invasive measurement of cardiac output in heart failure patients using a new foreign gas rebreathing technique. *Clin Sci.* 2002;102:247–252.

5. Agostoni P, Cattadori G, Apostolo A, et al. Noninvasive measurement of cardiac output during exercise by inert gas rebreathing technique: a new tool for heart failure evaluation. *J Am Coll Cardiol*. 2005;46:1779–1781.

6. McEniery CM, Yasmin, Hall IR, et al. Normal vascular aging: differential effects on wave reflection and aortic pulse wave velocity: the Anglo-Cardiff Collaborative Trial (ACCT). *J Am Coll Cardiol.* 2005;46:1753–1760.

7. Jugdaohsingh R, Anderson SHC, Lakasing L, Sripanyakorn S, Ratcliffe S, Powell JJ. Serum silicon concentrations in pregnant women and newborn babies. *BJN.* 2013;110:1–7.

8. Agatemor C, Beauchemin D. Matrix effects in inductively coupled plasma mass spectrometry: A review. *Analytica Chimica Acta.* 2011;706:66–83.

**Supplementary Table S1 – Differences in demographics and baseline variables for by gender**

| **Baseline variables** | **Males**  **(n=24)** | **Females**  **(n=24)** | **P-value** |
| --- | --- | --- | --- |
| Age, years | 28 ± 2 | 32 ± 2 | 0.14 |
| Ethnicity (Caucasian) | 22/24 | 23/24 | - |
| Body weight, kg | 74.3 ± 2.5 | 64.1 ± 1.5 | **< 0.001** |
| Height, cm | 178 ± 2 | 165 ± 2 | **< 0.001** |
| BMI, kg ms^-2^ | 22.7 (21.6 – 24.0) | 23.9 (22.1 – 25.4) | 0.29 |
|  |  |  |  |
| **Haemodynamic variables** |  |  |  |
| Seated SBP, mmHg | 123 ± 2 | 116 ± 2 | **0.004** |
| Seated DBP, mmHg | 67 ± 2 | 72 ± 2 | 0.45 |
| Seated MAP, mmHg | 90 ± 2 | 90 ± 2 | 0.89 |
| Seated HR, BPM | 71 ± 2 | 73 ± 2 | 0.32 |
| Supine SBP, mmHg | 122 ± 2 | 115 ± 2 | **0.03** |
| Supine DBP, mmHg | 67 ± 2 | 72 ± 2 | **0.04** |
| Supine MAP, mmHg | 87 ± 2 | 87 ± 2 | 0.99 |
| Supine HR, BPM | 64 ± 2 | 67 ± 2 | 0.37 |
| 24-hr MAP | 95 ± 2 | 91 ± 1 | **0.02** |
| 24-hr SBP | 122 ± 2 | 114 ± 1 | **< 0.001** |
| 24-hr DBP | 74 ± 2 | 72 ± 1 | 0.43 |
| Night-time SBP, mmHg | 113 ± 2 | 107 ± 1 | **0.01** |
| Night-time DBP, mmHg | 66 ± 2 | 64 ± 1 | 0.44 |
| Night-time MAP, mmHg | 88 ± 2 | 83 ± 1 | 0.05 |
| Augmentation index, % | 2.8 ± 2.0 | 15.0 ± 2.6 | **0.003** |
| PWV m/s | 5.3 (4.8 – 5.7) | 5.2 (4.8 – 5.8) | 0.61 |
| Cardiac output, litres/min | 6.3 ± 0.3 | 5.6 ± 0.3 | 0.06 |
| Stroke volume, ml | 101.9 ± 4.7 | 79.6 ± 3.6 | **< 0.001** |
| PVR, dynes s^-1^ cm^-5^ | 1156 (982.3 – 1324) | 1183(1032 – 1690) | 0.22 |
| **Biochemical variables** |  |  |  |
| eGFR, ml/min/1.72m^2^ | 114.3 (107.7 – 124.4) | 97.2 (88.4 - 117.2) | **0.01** |
| Renin, mU/l | 18.0 (13.0 – 25.5) | 9.0 (7 – 14) | **< 0.001** |
| Aldosterone, pmol/l | 166.0 (110 – 194) | 110.5 (69.0 – 279.5) | 0.27 |
| Plasma VEGF C, pg/ml | 542.7 (412.5 - 842.5) | 644 (469.7 - 1172.0) | 0.22 |
| sFlt-4, pg/ml | 9.1 ± 0.9 | 10.1 ± 1.0 | 0.45 |
| 24-hr Urine Na^+^ | 102.0 ± 12.4 | 84.9 ± 10.1 | 0.31 |
| 24-hr Urine K^+^ | 75.0 ± 8.9 | 64.2 ± 4.4 | 0.29 |
| 24-hr Urine Cl^-^ | 115.4 ± 12.7 | 101.5 ± 12.8 | 0.45 |

Normally distributed data presented as mean ± SEM. Non-normally distributed data are presented as median and IQR. The values for males and females was analyzed using the unpaired t test with normally distributed data and Man Whitney test for non-normal data. P value < 0.05 taken to be significant.

**Supplementary Table S2: Differences in skin biochemical responses to placebo vs. slow sodium in women according to contraceptive (OCP) use.**

| **Variables** | **No contraceptive use**  **n = 14** | | | **Contraceptive use**  **n = 10** | | |
| --- | --- | --- | --- | --- | --- | --- |
|  | **Placebo** | **Slow sodium** | **P-value** | **Placebo** | **Slow sodium** | **P-value** |
| **Skin Na^+^, mg/g** | 2.15 ± 0.07 | 2.15 ± 0.05 | 0.92 | 2.16 ± 0.07 | 2.12 ± 0.07 | 0.92 |
| **Skin K^+^, mg/g** | 0.65 ± 0.03 | 0.62 ± 0.02 | 0.43 | 0.73 ± 0.05 | 0.70 ± 0.04 | 0.50 |
| **Skin Na^+^:K^+^** | 3.37 ± 0.12 | 3.53 ± 0.13 | 0.43 | 3.04 ± 0.17 | 3.12 ± 0.20 | 0.69 |

Normally distributed data presented as mean ± SEM. The change between placebo and slow sodium for both groups was analyzed using the student’s paired t test. P value < 0.05 taken to be significant.

10 women were on contraceptive treatment, of which 6 were on the combined pill and 4 were on progesterone-only pill or progesterone implants. Contraceptive treatment did not seem to affect the skin Na^+^ or skin Na^+^:K^+^ response to dietary salt loading. Women on contraceptive treatment had higher skin K^+^ levels post slow sodium (p = 0.03) but not post placebo (p = 0.11) compared with females not on contraceptive treatment. Consequently, females on contraceptive treatment had a trend for lower skin Na^+^:K^+^ levels post placebo (p = 0.05) and post slow sodium (p = 0.09) compared with females not on contraceptive treatment.

**Supplementary Table S3 – Differences in demographics and baseline variables for women according to contraceptive use**

| **Baseline variables** | **No contraceptive**  **(n=14)** | **On contraceptive**  **(n=10)** | **P-value** | |  |
| --- | --- | --- | --- | --- | --- |
| Age, years | 33 ± 3 | 30 ± 2 | 0.55 | | |
| Ethnicity (Caucasian) | 13/14 | 10/10 | - | | |
| Body weight, kg | 65.0 ± 2.2 | 62.9 ± 2.0 | 0.52 | | |
| Height, cm | 163 ± 2 | 166 ± 1 | 0.31 | | |
| BMI, kg ms^-2^ | 24.4 ± 0.7 | 22.8 ± 0.8 | 0.14 | | |
| **Haemodynamic variables** |  |  |  | | |
| Seated SBP, mmHg | 117 ± 2 | 114 ± 4 | 0.51 | | |
| Seated DBP, mmHg | 75 ± 2 | 76 ± 2 | 0.80 | | |
| Seated MAP, mmHg | 89 ± 2 | 89 ± 3 | 0.91 | | |
| Seated HR, BPM | 75 ± 3 | 71 ± 2 | 0.36 | | |
| Supine SBP, mmHg | 115 ± 3 | 115 ± 4 | 0.94 | | |
| Supine DBP, mmHg | 72 ± 2 | 72 ± 3 | 0.99 | | |
| Supine MAP, mmHg | 87 ± 2 | 86 ± 3 | 0.96 | | |
| Supine HR, BPM | 67 ± 2 | 66 ± 2 | 0.67 | | |
| 24-hr MAP | 91 ± 1 | 92 ± 2 | 0.62 | | |
| 24-hr SBP | 114 ± 2 | 114 ± 2 | 0.99 | | |
| 24-hr DBP | 72 ± 1 | 73 ± 1 | 0.59 | | |
| Night-time SBP, mmHg | 106 ± 2 | 108 ± 2 | 0.50 | | |
| Night-time DBP, mmHg | 62 ± 2 | 66 ± 2 | 0.59 | | |
| Night-time MAP, mmHg | 82 ± 1 | 85 ± 2 | 0.13 | | |
| Augmentation index, % | 17.8 ± 3.4 | 11.0 ± 3.8 | 0.21 | | |
| PWV m/s | 5.4 (4.9 – 5.9) | 5.0 (4.5 – 5.8) | 0.19 | | |
| Cardiac output, litres/min | 5.3 ± 0.3 | 6.0 ± 0.4 | 0.17 | | |
| Stroke volume, ml | 75.3 ± 4.1 | 85.7 ± 6.2 | 0.16 | | |
| PVR, dynes s^-1^ cm^-5^ | 1265 (1011 – 1880) | 1183(1046 – 1348) | 0.22 | | |
| **Biochemical variables** |  |  |  | | |
| eGFR, ml/min/1.72m^2^ | 95.1 (88.7 – 115.2) | 103.7 (87.1 – 151.1) | | 0.73 | |
| Renin, mU/l | 14.1 ± 2.1 | 7.0 ± 0.8 | | **0.02** | |
| Aldosterone, pmol/l | 113 (69 – 247) | 87 (70 – 385) | | 0.83 | |
| Plasma VEGF C, pg/ml | 831.9 ± 116.1 | 840.3 ± 195.9 | | 0.97 | |
| sFlt-4, pg/ml | 9.0 ± 1.3 | 11.8 ± 1.6 | | 0.19 | |
| 24-hr Urine Na^+^,mmol | 101.1 ± 14.7 | 65.4 ± 11.6 | 0.08 | | |
| 24-hr Urine K^+^,mmol | 61.6 ± 5.8 | 67.3 ± 6.9 | 0.53 | | |
| 24-hr Urine Cl^-^,mmol | 122.7 ± 19.0 | 76.1 ± 13.5 | 0.07 | | |

Normally distributed data presented as mean ± SEM. Non-normally distributed data are presented as mean and IQR. The values for males and females was analyzed using the unpaired t test with normally distributed data and Man Whitney test for non-normal data. P value < 0.05 taken to be significant.

**Table S4 – Differences in haemodynamic responses to placebo vs. slow sodium according to contraceptive use.**

| **Variables** | **No contraceptive (n=14)** | | | **On contraceptive (n=10))** | | |
| --- | --- | --- | --- | --- | --- | --- |
|  | **Placebo** | **Slow sodium** | **P-value** | **Placebo** | **Slow sodium** | **P-value** |
| Body weight, kg | 64.2 ± 2.3 | 64.8 ± 2.4 | 0.12 | 62.4 ± 2.0 | 63.2 ± 1.7 | **0.03** |
| **Office measurements** |  |  |  |  |  |  |
| Seated SBP, mmHg | 115 ± 2 | 116 ± 2 | 0.48 | 111 ± 3 | 111 ± 3 | 0.85 |
| Seated DBP, mmHg | 74 ± 2 | 74 ± 2 | 0.96 | 71 ± 2 | 72 ±2 | 0.63 |
| Seated MAP, mmHg | 88 ± 2 | 88 ± 2 | 0.68 | 85 ± 3 | 86 ± 2 | 0.50 |
| Seated HR, bpm | 77 ± 2 | 75 ± 3 | 0.42 | 69 ± 3 | 68 ± 3 | 0.55 |
| Supine SBP, mmHg | 111 ± 3 | 115 ± 3 | 0.15 | 111 ± 3 | 110 ± 2 | 0.79 |
| Supine DBP, mmHg | 68 ± 2 | 70 ± 2 | 0.33 | 68 ± 2 | 68 ± 1 | 0.64 |
| Supine MAP, mmHg | 83 ± 2 | 85 ± 2 | 0.18 | 82 ± 2 | 82 ± 2 | 0.74 |
| Supine HR, bpm | 67 ± 2 | 64 ± 2 | 0.31 | 62 ± 3 | 61 ± 3 | 0.58 |
| **Ambulatory BP, mmHg** |  |  |  |  |  |  |
| 24-hr SBP | 114 ± 1 | 118 ± 2 | **0.02** | 113 ± 2 | 118 ± 2 | **0.004** |
| 24-hr DBP | 71 ± 2 | 72 ± 2 | 0.54 | 71 ± 1 | 74 ± 1 | **0.03** |
| 24-hr MAP | 91 ± 2 | 93 ± 2 | 0.07 | 90 ± 1 | 94 ± 2 | **0.001** |
| Day SBP | 119 ± 2 | 121 ± 3 | 0.19 | 117 ± 2 | 121 ± 3 | 0.05 |
| Day DBP | 75 ± 3 | 75 ± 2 | 0.95 | 75 ± 1 | 77 ± 2 | 0.17 |
| Day MAP | 95 ± 2 | 96 ± 2 | 0.47 | 94 ± 2 | 97 ± 2 | **0.04** |
| Night SBP | 104 ± 2 | 109 ± 2 | **0.001** | 106 ± 2 | 111 ± 1 | **0.001** |
| Night DBP | 61 ± 2 | 64 ± 2 | 0.10 | 64 ± 1 | 67 ± 1 | 0.07 |
| Night MAP | 81 ± 2 | 85 ± 2 | **0.008** | 83 ± 2 | 87 ± 1 | **0.005** |
| **Central haemodynamics** |  |  |  |  |  |  |
| CSBP, mmHg | 95 ± 2 | 101 ± 3 | **0.01** | 95 ± 3 | 93 ± 3 | 0.44 |
| CDBP, mmHg | 69 ± 2 | 71 ± 2 | 0.62 | 69 ± 2 | 69 ± 2 | 0.96 |
| CMAP, mmHg | 82 ± 2 | 87 ± 2 | **0.01** | 83 ± 3 | 81 ± 2 | 0.44 |
| Augmentation index, % | 15.7 ± 3.6 | 19.1 ± 3.8 | 0.10 | 10.9 ± 4.9 | 8.1 ± 4.3 | 0.10 |
| PWV ms^-1^ | 5.6 ± 0.3 | 5.5 ± 0.2 | 0.91 | 5.0 ± 0.1 | 5.0 ± 0.1 | 0.95 |
| Cardiac output, litres/min | 5.5 ± 0.3 | 5.6 ± 0.3 | 0.46 | 5.8 ± 0.5 | 6.2 ± 0.5 | 0.43 |
| Stroke volume, ml | 82.8 ± 4.7 | 85.2 ± 4.6 | 0.42 | 90.4 ± 6.9 | 94.0 ± 5.7 | 0.51 |
| PVR, dynes s^-1^ cm^-5^ | 1268 ± 91 | 1278 ± 92 | 0.79 | 1229 ± 123 | 1119 ± 87 | 0.36 |

Normally distributed data presented as mean ± SEM. Student’s paired t-tests were applied to paired observations after placebo and slow sodium

**Supplementary Table S5- Correlations for 24-hr urine Na^+^:K^+^ and other parameters** **in men (n=24). Clinic MAP refers to supine brachial MAP. Correlation for urine Na^+^:K^+^ and stroke volume post placebo was not independent of age. Correlation for urine Na^+^:K^+^ and PVR post placebo was not independent of BMI.**

|  |  | **Clinic MAP** | **Stroke volume** | **PVR** | **Skin Na^+^:K^+^** |
| --- | --- | --- | --- | --- | --- |
| **Urine Na^+^:K^+^** | **Placebo** | r = 0.10  p = 0.66 | r = - 0.42  p = 0.046 | r = 0.51  p = 0.01 | r = 0.21  p = 0.34 |
|  | **Slow Na** | r = 0.10  p = 0.64 | r = - 0.22  p = 0.30 | r = 0.12  p = 0.59 | r = 0.08  p = 0.70 |

Correlation shown are Pearson’s correlation apart from PVR (Spearmans)

**Supplementary Table S6- Correlations for 24-hr urine Na^+^:K^+^ and other parameters in women (n=24).**

|  |  | **Clinic MAP** | **Stroke volume** | **PVR** | **Skin Na^+^:K^+^** |
| --- | --- | --- | --- | --- | --- |
| **Urine Na^+^:K^+^** | **Placebo** | r = - 0.18  p = 0.40 | r = - 0.01  p = 0.96 | r = - 0.16  p = 0.46 | r = - 0.33  p = 0.11 |
|  | **Slow Na** | r = - 0.17  p = 0.42 | r = - 0.01  p = 0.96 | r = - 0.27  p = 0.20 | r = 0.10  p = 0.63 |

Correlation shown are Pearson’s correlations.

**Supplementary Table S6: Skin Na^+^ and % water content for pilot study and samples obtained from breast reduction surgery taken under general anaesthetic.**

| **Variables** | **Pilot skin**  **n = 37** | **Breast reduction skin**  **n = 17** | **P-value** |
| --- | --- | --- | --- |
| **Skin Na^+^, mg/g** | 2.15 ± 0.04 | 2.25 ± 0.06 | 0.18 |
| **% water** | 60.9 ± 1.3 | 62.3 ± 1.6 | 0.29 |
| **Age, years** | 39 ± 3 | 43 ± 4 | 0.38 |

Normally distributed data presented as mean ± SEM. The differences between groups was analyzed using the unpaired t-test. P value < 0.05 taken to be significant.

**Supplementary Table S7: Running conditions used for ICP-OES.**

| **Analytical Conditions** | **Na:K** |
| --- | --- |
| **RF power (W)** | 1000 |
| **Plasma gas (L min^-1^)** | 12 |
| **Sheath gas (L min^-1^)** | 2 |
| **Auxiliary gas (L min^-1^)** | 0.0 |
| **Speed pump (rates min^-1^)** | 10 |
| **Nebulizer gas flow rate (L/min^-1^)** | 0.73 |
| **Nebulizer pressure (bar)** | 2.75 |
| **Number of replicates** | 3 |

**Supplementary Table S8: Peak profile measurement parameters**

| **Element** | **Na** | **K** | **Sr** |
| --- | --- | --- | --- |
| **Wavelength (nm)** | 588.995 | 766.490 | 346.446 |
| **Number of points** | 21 | 11 | 9 |
| **Integration time (s)** | 0.5 | 0.5 | 0.5 |
| **Increments (nm)** | 0.001 | 0.002 | 0.002 |
| **Photomultiplicator tube voltage (V)** | 911 | 990 | 750 |
| **Photomultiplicator tube gain (%)** | 100 | 100 | 100 |
| **Points used** | 5 | 5 | 5 |
| **Number of replicates** | 3 | 3 | 3 |
| **Calculation mode** | Gauss | Gauss | Gauss |

**Supplementary Figure S1 – Study design showing 4-week double blind crossover design.**

**
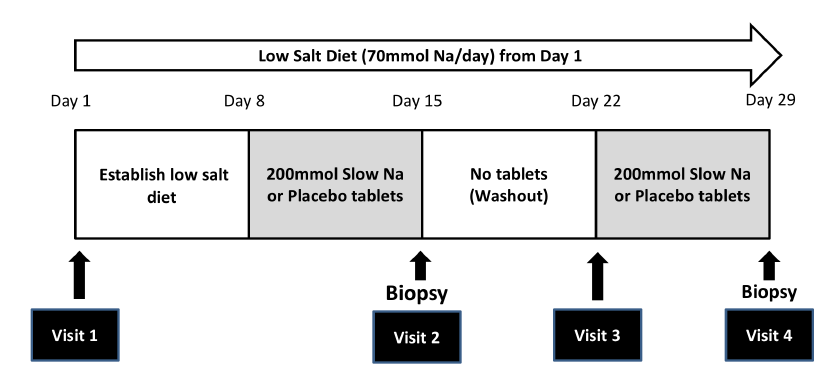
**

**Supplementary Figure S2: Technique used for injection of local anaesthetic.**

**
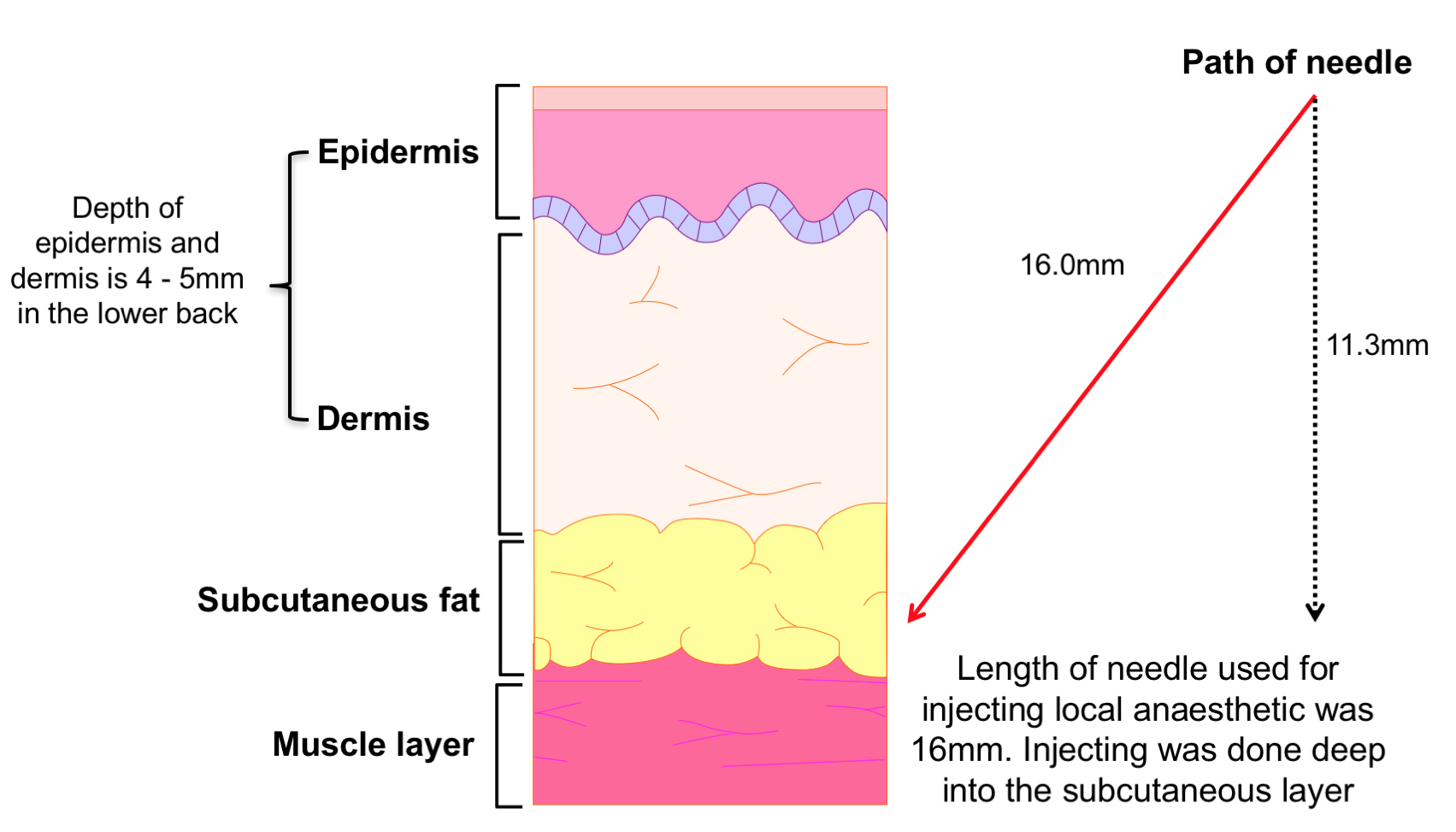
**

**Supplementary Figure S3: Technique used for skin analysis by ICPOES.**

**
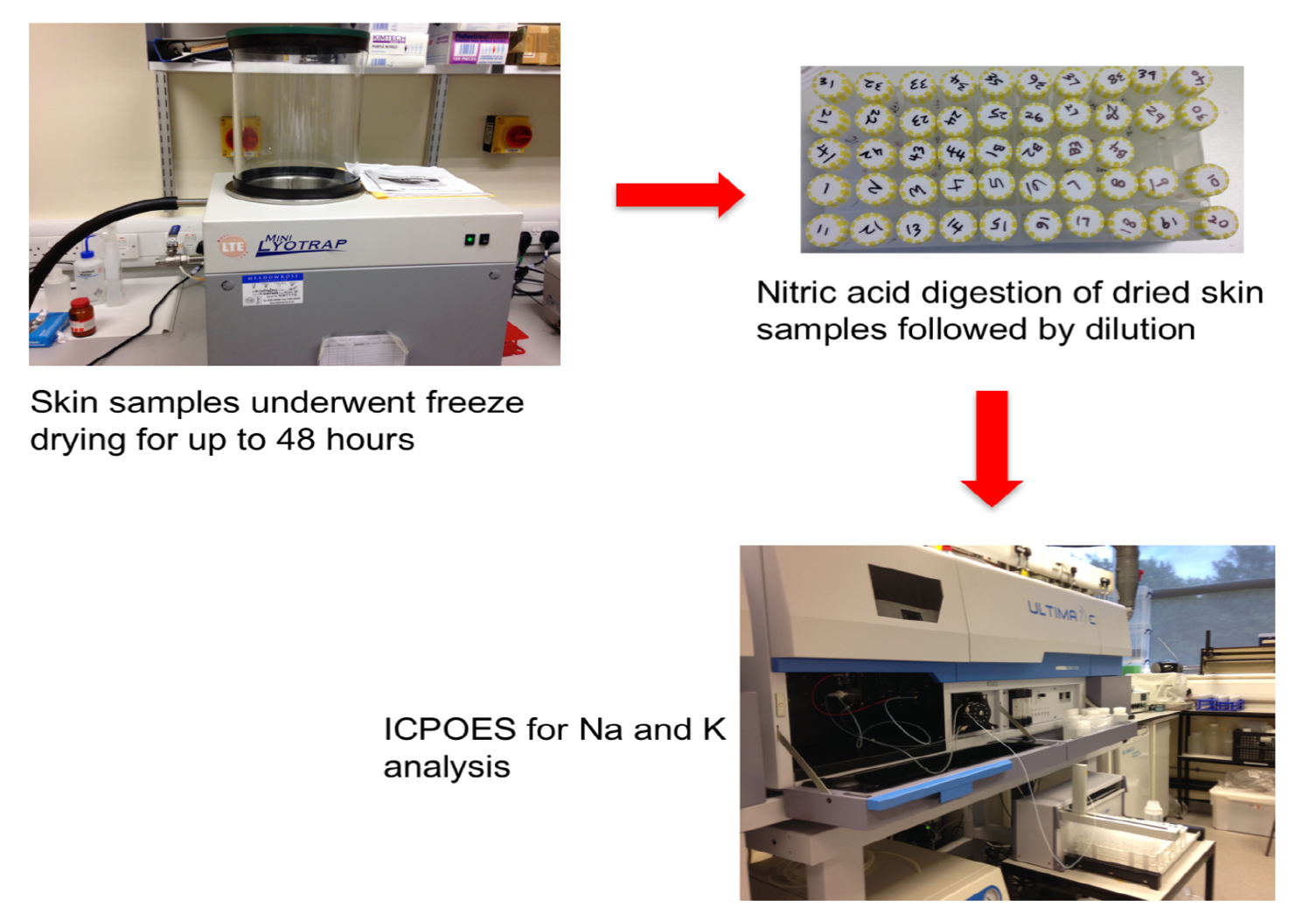
**

**Supplementary Figure S4: Comparison of Skin Na^+^:K^+^ between male participants who received conventional local anaesthetic (n = 14) and sodium-free anaesthetic (n = 10) in the main study.**

**
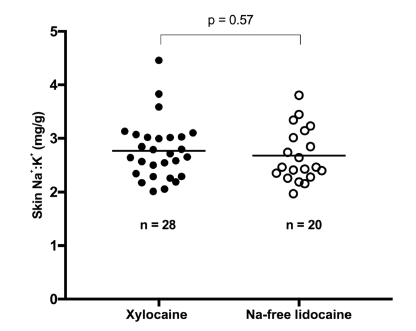
**

Skin Skin Na^+^:K^+^ values post placebo and slow sodium were pooled for each group. Conventional local anaesthetic (Xylocaine 1% with adrenaline, Astra Zeneca) contains Na while Na-free lidocaine (1% lidocaine with 3.5% Dextrose, Tayside Pharmaceuticals) was confirmed by ICP-OES to be Na-free. As seen in Figure 4S, no significant difference to suggest Na contamination was evident.

**Supplementary Figure S5: Changes in skin Na^+^:K^+^ ratios between placebo and slow sodium phases. A. Men (n=24), B. Women not on contraceptive treatment (n=14) C. women on contraceptive treatment (n=10).**


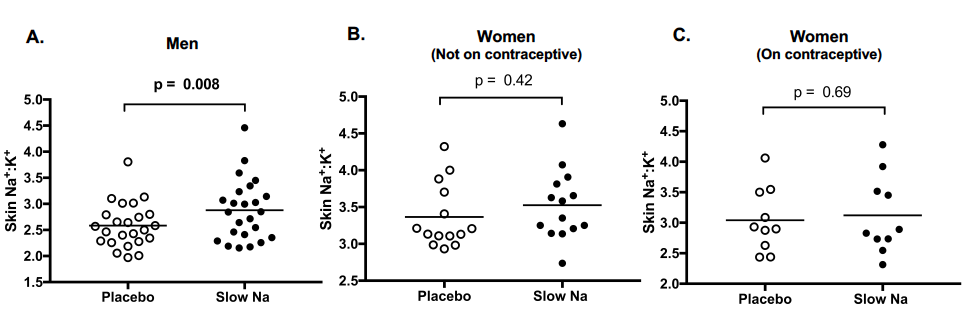


**Supplementary Figure S6: Correlations for skin Na^+^:K^+^ and haemodynamic parameters in women (n=24).**

Correlation seen with PVR post salt (Fig F) was not significant after correction for age.


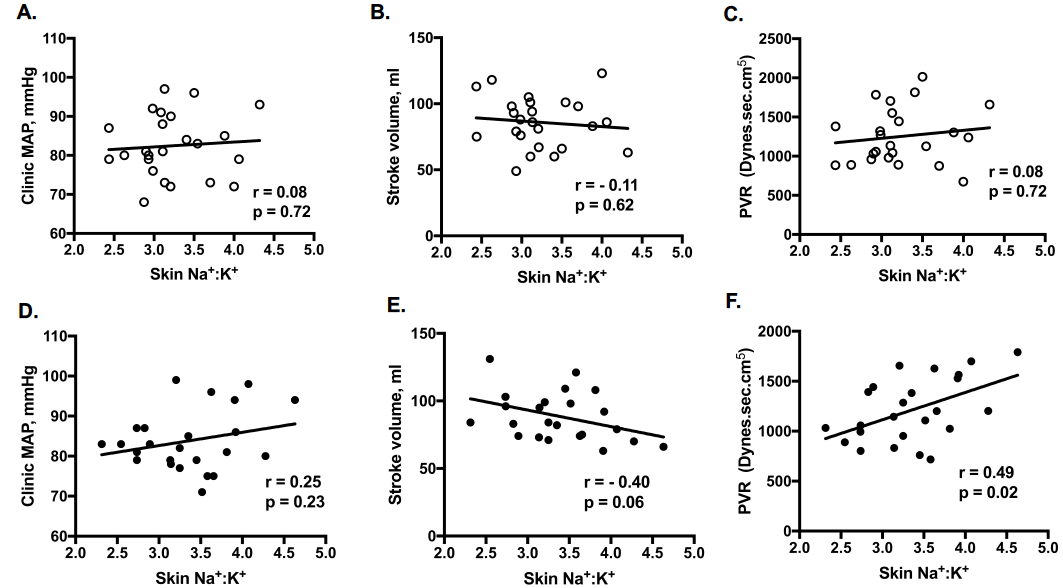

Supplement: Supplementary file 1 [file hyp-70-0930-s001.docx]
